# Predictors of Treatment with Duloxetine or Venlafaxine XR among Adult Patients Treated for Depression in Primary Care Practices in the United Kingdom

**DOI:** 10.1155/2012/815363

**Published:** 2012-06-07

**Authors:** Nianwen Shi, Emily Durden, Amelito Torres, Zhun Cao, Michael Happich

**Affiliations:** ^1^Thomson Reuters, Cambridge, MA 02140, USA; ^2^Thomson Reuters, Washington, DC 20008, USA; ^3^General Electric Healthcare, 2 Bethesda Metro Center, Bethesda, MD 20814, USA; ^4^Eli Lilly and Company, Health Technology Appraisal Group, 61352 Bad Homburg, Germany

## Abstract

*Background.* Knowledge about real-world use of duloxetine and venlafaxine XR to treat depression in the UK is limited. *Aims.* To identify predictors of duloxetine or venlafaxine XR initiation. *Method.* Adult depressed patients who initiated duloxetine or venlafaxine XR between January 1, 2006 and September 30, 2007 were identified in the UK's General Practice Research Database. Demographic and clinical predictors of treatment initiation with duloxetine and venlafaxine XR were identified using logistic regression. *Results.* Patients initiating duloxetine (*n* = 909) were 4 years older than venlafaxine XR recipients (*n* = 1286). Older age, preexisting unexplained pain, respiratory disease, and pre-period use of anticonvulsants, opioids, and antihyperlipidemics were associated with increased odds of initiating duloxetine compared to venlafaxine XR. Pre-period anxiety disorder was associated with decreased odds of receiving duloxetine. *Conclusion.* Initial treatment choice with duloxetine versus venlafaxine XR was primarily driven by patient-specific mental and medical health characteristics. General practitioners in the UK favor duloxetine over venlafaxine XR when pain conditions coexist with depression.

## 1. Introduction

Major depressive disorder (MDD) is a mood disorder characterized by persistent feelings of sadness, a pervasive low mood, diminished ability to experience pleasure, and cognitive symptoms such as difficulty concentrating and impaired memory. In addition to the array of emotional and cognitive symptoms of depression, people with depression often have physical symptoms that do not respond well to treatment, such as frequent headaches, digestive problems, and chronic pain. Taken together, the symptoms of depression can lead to significant impairments in cognitive, physical, and social functioning. In the UK, the prevalence of depression is 2.6% among those aged 16–74, with a slightly higher rate among females [1]. A total of 1.24 million people were estimated having depression in England in 2007 [2]. A variety of pharmacologic treatments are available to alleviate the symptoms of depression, including tricyclic antidepressants (TCAs), monoamine oxidase inhibitors (MAOIs), selective serotonin reuptake inhibitors (SSRIs), and dopamine reuptake inhibitors. Serotonin norepinephrine reuptake inhibitors (SNRIs), a relatively new class of antidepressant medications, have a selective enhancing effect on both serotonin and norepinephrine neurotransmission. Introduced in 1995 in the UK, venlafaxine XR is a SNRI indicated for major depressive disorder, generalized anxiety disorder, social anxiety disorder, and panic disorder. Duloxetine, marketed in 2005, is approved for treatment of major depressive disorder, diabetic peripheral neuropathic pain, and generalized anxiety disorder.

In recent years, studies have evaluated the efficacy of SNRIs compared to SSRIs as well as the use of SNRIs in patients with severe depression [3–6]. Efficacy and cost effectiveness among duloxetine and venlafaxine XR have also been compared [7–10]. Using the Global Benefit-Risk (GBR) assessment, Perahia et al. [10] reported similar overall risk-benefit profiles for duloxetine and venlafaxine XR from two randomized controlled studies of patients receiving either duloxetine 60 mg/day or venlafaxine XR 150 mg/day. Although no significant differences in the major efficacy measures were observed, a higher percentage of patients in the venlafaxine arm completed 12 weeks of treatment. Duloxetine-treated patients were more likely to report nausea as a treatment-emergent adverse event than venlafaxine-treated patients, and venlafaxine-treated patients were more likely to suffer from discontinuation-emergent adverse events during the taper period than were duloxetine-treated patients. Additionally, venlafaxine-treated patients were more likely to suffer from discontinuation-emergent adverse events during the taper period than were duloxetine-treated patients. The venlafaxine arm also had a higher rate of sustained, elevated systolic blood pressure than the duloxetine arm during the fixed dosing period. Currently, both drugs are recommended as second-line treatment for depression by the National Institute for Health and Clinical Excellence (NICE) in the UK [11]. In response to postmarketing reports suggesting potential cardiovascular toxicity of venlafaxine XR, the Medicines and Healthcare products Regulatory Agency (MHRA) in the UK issued a warning for patients with heart disease treated with venlafaxine XR in 2006. Venlafaxine is contraindicated in patients with high risk of cardiac ventricular arrhythmia and uncontrolled hypertension [12].

The use of duloxetine monotherapy versus venlafaxine and other antidepressants among patients with MDD in the real-world setting has been examined in several retrospective claim-based studies. Based on the Veterans Health Administration in the United States (US), Shi et al. [13] suggested that prior opioid use, moderate-to-severe pain, and substance abuse were predictors of duloxetine initiation comparing to nonduloxetine antidepressants. Using US administrative data from 2004 to 2006, Ye et al. [14] evaluated the predictors of treatment with duloxetine and venlafaxine XR. Older age, previously treated with SSRIs, TCAs, other antidepressants, anticonvulsants, atypical antipsychotics, analgesics, hypnotics, muscle relaxants, stimulants, or antihistamines, presence of sleeping disorder, receiving 3 or more different pain medications in the pre-period, being treated by psychiatrists, were found as significant predictors of duloxetine initiation. However, to our best knowledge, there is no similar study examining the impact of patient characteristics on treatment choice with duloxetine or venlafaxine XR in clinical practice outside of the US. To address this knowledge gap, this study analyzed the demographic and clinical characteristics of patients with depression in the UK who initiated pharmacologic treatment with duloxetine or venlafaxine XR and identified predictors of treatment choice using multivariate regression.

## 2. Materials and Methods

### 2.1. Data Sources

Study patients were identified from the General Practice Research Database (GPRD), which contains detailed, and deidentified medical information on enrollees in the UK's primary care system. The study sample was extracted in February, 2009, at a time when the GPRD had over 8.9 million research-useable patients, including 3 million active enrollees from nearly 350 primary care practices throughout the UK. These represented a 5.5% subsample generalizable to the entire population of patients in UK's primary care health system at the time. The database provides comprehensive, longitudinal data from real-world clinical practices including symptoms, diagnoses, medication orders, procedures, tests, immunizations, and patient demographic characteristics and is widely used in health outcomes and epidemiological research. Because our study did not involve the collection, use, or transmittal of individually identifiable data, Institutional Review Board (IRB) oversight was not required.

### 2.2. Patient Selection Criteria

**Figure 1:**
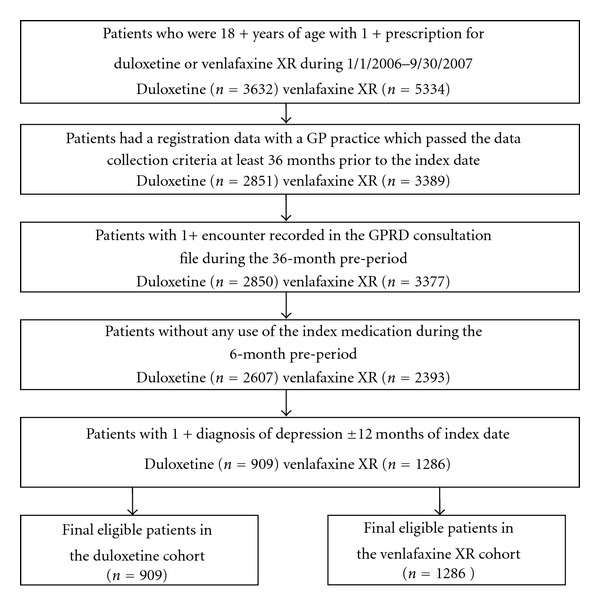
Patient Selection.

Patients with at least one prescription order for duloxetine or venlafaxine XR between January 1, 2006 and September 30, 2007 were identified from the GPRD. All eligible patients were required to be 18 years or older on the date of the index prescription. Patients were required to be registered with a General Practice (GP) during the entire study period. GPs must have reached the minimum required standards for recording data within the practice in five areas including registration, prescribing, death, female health, and referrals at the beginning of the study period. In addition, at least one patient encounter, face-to-face or non-face-to-face, was required with a GP in the 36 months before the index prescription. Patients were required to have no prescriptions for duloxetine or venlafaxine XR in the six months prior to the index date and have at least one diagnosis of depression in the 12 months prior to or following the index prescription order. Patients meeting the inclusion criteria were classified into one of two treatment cohorts based on their index SNRI treatment regimen: (1) duloxetine or (2) venlafaxine XR.  Figure 1 displays the impact of each inclusion criterion on the final sample.

The date of the first prescription order for duloxetine or venlafaxine XR between January 1, 2006 and September 30, 2007 was set as the index date. The 36 months preceding the index date constituted the pre-period (and included the clean period of six months for the index SNRI). Preexisting medical conditions as potential predictors of index SNRI treatment were evaluated during this period. Pre-period use of medications as potential predictors of the index treatment was evaluated during the 12 months immediately preceding the index date. A longer evaluation period was applied to medical conditions than medication use because diagnoses, especially those for chronic conditions, may not be entered in the GP records each time a patient has an encounter with a practitioner. The longer evaluation period, therefore, increased the chances of capturing all diagnoses of interest.

### 2.3. Dependent Variable

The objective of this study was to identify predictors of treatment initiation with duloxetine or venlafaxine XR among patients with depression. The dependent variable was a dichotomous indicator of receiving the index treatment of duloxetine versus venlafaxine XR. Patient demographic and baseline clinical characteristics were examined to determine the factors with significant impact on the odds of initiating duloxetine or venlafaxine XR.

### 2.4. Covariates

Patient demographic characteristics included age at index date and gender. A series of binary flags was created to denote the presence of the following psychiatric diagnoses appearing in the 36-month pre-period: anxiety disorders, attention deficit hyperactivity disorder, schizophrenia, bipolar disorder, and alcohol/drug dependence. Additional binary flags were created to denote the following physical health diagnoses appearing in the 36-month pre-period: circulatory, respiratory, or digestive system diseases; sleep disorders; diabetes; diabetic peripheral neuropathy (DPN); fibromyalgia; osteoarthritis; chronic low-back pain; unexplained pain which included joint, musculoskeletal, muscle, noncardiac chest, abdominal, and other pain. Medical and psychiatric conditions were identified through Read/OXMIS codes recorded in the GPRD Clinical and Referral files. A list of search terms for each of the conditions of interest was identified. Text string searches were conducted to identify relevant Read/OXMIS codes, which were reviewed jointly by researchers and clinical coding specialists. Code ranges with close proximity to the identified Read/OXMIS were also reviewed to ensure that all relevant codes were captured.

Prescription orders appearing in the 12-months pre-period for medications used to treat a variety of mental health conditions were identified, including antidepressants (SNRIs, SSRIs, TCAs, MAOIs, and other antidepressants), anxiolytics/hypnotics/muscle relaxants, antipsychotics, stimulants, antimanics, and anticonvulsants. Similarly, prescription orders for medications used to treat various physical health conditions during the same period were identified, including cardiovascular medications, thyroid hormones, antidiabetic medications, gastrointestinal medications, respiratory medications, and analgesics (opioids, nonopioids, and migraine medications).

In addition to the preexisting medical conditions and pre-index medication use, indicators for any inpatient and accident and emergency (AE) event in the 12 months preceding the index date were created. Inpatient events were determined by the presence of Read/OXMIS codes and/or consultation types indicating inpatient admission or discharges recorded in the GPRD consultation, clinical, and referral files. Similarly, AE events were identified by the presence of relevant Read/OXMIS codes, consultation type, or provider specialties indicative of AE services.

### 2.5. Statistical Analysis

#### 2.5.1. Univariate Analysis

Frequency distributions, means, and standard deviations were used to describe the demographic and pre-period clinical characteristics of the study population. Differences between treatment cohorts in demographic and clinical characteristics were evaluated using chi-square tests for categorical variables and *t*-tests for continuous variables. Differences with *P* < 0.05 were considered statistically significant.

#### 2.5.2. Multivariate Analysis

Multivariate logistic regression was used to determine significant predictors of index SNRI treatment. Patient demographic characteristics, pre-period medical conditions, and pharmacological treatment common among patients with depression, pre-period healthcare utilization, as listed under the “Covariates” section, were included in the model. The venlafaxine cohort served as the reference group. The odds ratio (OR) for patients initiating treatment with duloxetine was computed as the exponential of the logistic regression coefficient. *P* values for all ORs were considered statistically significant when *P* < 0.05. Stata MP 11 software was used in the multivariate analysis.

## 3. Results and Discussion

### 3.1. Demographic Characteristics

A total of 2,195 patients were identified for the analysis with an average age of 47.2 years. Approximately 41% were prescribed duloxetine at index, while 59% received venlafaxine XR. Women were overrepresented in both treatment cohorts, but no significant difference was observed in the gender distribution between groups (67.7%/duloxetine, 64.5%/venlafaxine XR, *P* = 0.120). On average, patients treated with duloxetine were 4 years older than patients treated with venlafaxine XR (49.6 ± 16.5 years versus 45.5 ± 16.1 years; *P* < 0.001). Higher mean age in the duloxetine cohort was driven by a higher percentage of patients in the age 65+ group and fewer patients in 18–34 and 35–44 age ranges (Table 1).

### 3.2. Clinical Characteristics

Table 2 presents the prevalence of general physical health and psychiatric conditions as well as healthcare utilization in the pre-period among patients in the two treatment cohorts. Patients treated with duloxetine at index had higher prevalence of a variety of physical health conditions in the pre-period compared to patients treated with venlafaxine XR, including diseases of the respiratory system (7.3 percentage points higher; *P* < 0.001), diseases of the circulatory system (6.9 percentage points higher, *P* < 0.001), diseases of the digestive system (5.5 percentage points higher, *P* = 0.007), and diabetes (3.8 percentage points higher, *P* < 0.001). Additionally, patients treated with duloxetine had higher prevalence of chronic low-back pain (1.2 percentage points higher, *P* = 0.002) and unexplained pain (11.9 percentage points higher, *P* < 0.001) than patients treated with venlafaxine XR. With the exception of anxiety disorders, no significant differences were observed between the two groups in the prevalence of the mental health conditions evaluated. Patients treated with duloxetine had a lower pre-period rate of anxiety disorders compared to venlafaxine XR recipients (3.6 percentage points lower, *P* = 0.049).

During the 12-month pre-period, 20.7% of patients treated with duloxetine and 17.4% of patients treated with venlafaxine XR had an inpatient event, but the difference was not statistically significant (*P* = 0.054). Compared to patients initiating treatment with venlafaxine XR, patients treated with duloxetine had a higher rate of AE events (22.8% versus 19.1%, *P* = 0.034).

Table 3 displays the rates of pre-period medication use by patients in the duloxetine and venlafaxine XR groups. During the 12-month pre-index period, patients in both treatment groups had similar exposure to antidepressant treatment but some differences were observed. Approximately three-quarters of duloxetine and venlafaxine XR patients were treated with an SSRI in the 12-month pre-index period, and about one-quarter was prescribed a TCA or other antidepressant type. In addition, 11%–13% had no evidence of antidepressant treatment. Compared to venlafaxine XR recipients, a higher proportion of patients treated with duloxetine at index received treatment with a TCA (25.1% versus 21.2%, *P* = 0.034) or a nonindex SNRI antidepressant (2.8% versus 0.8%, *P* = 0.036) in the pre-period.

Patients treated with duloxetine had a higher rate of use anticonvulsants than patients treated with venlafaxine XR (4.3 percentage points higher, *P* < 0.001). No statistically significant differences were observed in the utilization of other psychiatric medications, including anxiolytics/hypnotics/muscle relaxants, antipsychotics, stimulants, and antimanics. With regard to analgesic use, patients treated with duloxetine at index had a higher rate of receiving opioids (12.5 percentage points higher, *P* < 0.001) and nonopioids (13.1 percentage points higher, *P* < 0.001) in the pre-period than patients treated with venlafaxine XR. Consistent with the pattern observed for the prevalence of select medical conditions in the pre-period, the use of cardiovascular medications (12.2 percentage points higher, *P* < 0.001), thyroid hormones (2.3 percentage points higher, *P* = 0.029), antidiabetic medications (4.2 percentage points higher, *P* < 0.001), and GI medications (8.6 percentage points higher, *P* < 0.001) was significantly higher among patients prescribed duloxetine compared to patients prescribed venlafaxine XR at index.

### 3.3. Multivariate Results: Predictors of Index SNRI Treatment Type

**Figure 2:**
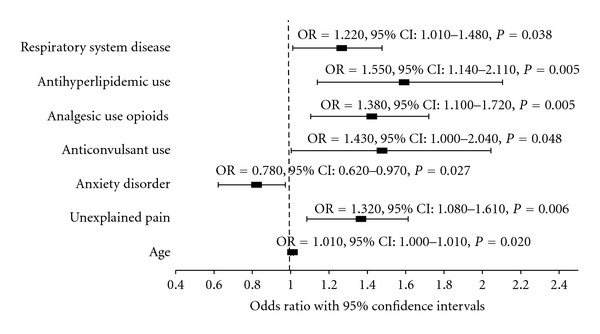
Logistic regression predicting duloxetine or venlafaxine XR at index. (Venlafaxine XR served as the reference group in the model. Only predictors with statistical significance are displayed.)

Significant results of the multivariate logistic regression model evaluating predictors of index SNRI treatment are presented in Figure 2. Unexplained pain in the pre-period was associated with 32% higher odds of receiving duloxetine (OR = 1.32, 95% CI: 1.08–1.61, *P* = 0.006). Respiratory system disease in the pre-period (OR = 1.22, 95% CI: 1.01–1.48, *P* = 0.038), pre-period use of anticonvulsants (OR = 1.43, 95% CI: 1.00–2.04, *P* = 0.048), opioid analgesics (OR = 1.38, 95% CI: 1.10–1.72, *P* = 0.005), and antihyperlipidemics (OR = 1.55, 95% CI: 1.14–2.11, *P* = 0.005) was also associated with significantly higher odds of initiating treatment with duloxetine. Finally, the presence of anxiety disorders in the pre-period was associated with a 22% lower odds of receiving treatment with duloxetine compared to venlafaxine XR at index (OR = 0.78, 95% CI: 0.62–0.97, *P* = 0.027). Age also significantly predicted treatment choice as an increase of 1 year in age was associated with 0.8% increase in the odds of receiving duloxetine (OR = 1.01, 95% CI: 1.00–1.01, *P* = 0.020).

### 3.4. Discussion

In 2009, NICE issued updated treatment guidelines for depression by reviewing the clinical efficacy, side-effect profiles, tolerability, discontinuation symptoms, safety in overdose, and cost effectiveness of various types of antidepressants. SSRIs are recommended as the first-line therapy for depression, and a variety of other antidepressant classes, including the SNRIs. Duloxetine and venlafaxine XR are suggested as second-line treatment for patients who have no or minimal response within 2–4 weeks following first-line treatment initiation [11]. Drawing from real-world experience in the UK's primary care health system among patients who received pharmacological treatment for depression, our study examined a wide array of pretreatment characteristics as predictors of duloxetine and venlafaxine XR treatment. Consistent with the NICE guidelines, the majority of patients in our study were treated with an SSRI in the 12 months prior to receiving duloxetine or venlafaxine XR. Duloxetine recipients were, on average 4 years older, and had higher prevalence of select clinical conditions including unexplained pain, diabetes, and diseases of the respiratory, circulatory, and digestive systems in the pre-period than patients who received venlafaxine XR. Patients treated with duloxetine also had higher rates of receiving medications such as cardiovascular medications, thyroid hormones, antidiabetic medications, and gastrointestinal medications, as well as a higher rate of AE in the pre-period than patients treated with venlafaxine XR. In contrast, patients treated with venlafaxine XR at index had a higher rate of anxiety disorders than those treated with duloxetine.

In our study, depressed patients with unexplained pain and/or analgesic opioid use had higher odds of receiving duloxetine than venlafaxine XR. This finding was consistent with the recent published Shi et al. [13] study in the US, in which opioid use and moderate-to-severe pain were identified as predictors of duloxetine monotherapy. Similarly, Ye et al. [14] reported that patients were more likely to receive duloxetine if they had previously received analgesics or used ≥3 unique pain medications in the pre-period. Pain often coexists with depression, and the improvement of pain symptoms is an important consideration when deciding among treatments for depression. The incidence rates of DPN in both the duloxetine and venlafaxine XR study cohorts were very low, and the rates for other pain conditions such as fibromyalgia, osteoarthritis, and chronic low-back pain were less than 7%. But unexplained pain was reported by 45% of duloxetine and 34% of venlafaxine XR patients in the pre-period. Venlafaxine XR has no indication for pain conditions, but duloxetine is indicated for DPN in addition to MDD in the UK and has documented effectiveness in managing pain in depression [15–18]. Using data from two pooled clinical trials of 251 duloxetine users and 261 placebo users, Fava et al. [15] reported that duloxetine significantly reduced pain severity among MDD patients compared to placebo, and pain reduction was associated with higher remission rates of MDD. In another placebocontrolled clinical trial, Brecht et al. [16] reported higher remission rate of MDD and better response rates in pain among duloxetine-treated MDD patients with at least moderate pain. In a study of MDD patients who had less-than-optimal responses to SSRI and switched to duloxetine, painful physical symptoms (PPSs) were significantly improved following the switch [17]. Clinical effectiveness of duloxetine in the short and long-term treatment of PPS was also evident among patients with generalized anxiety disorders [18]. Consistent with these studies findings, our study suggested that general practitioners in the UK were more likely to choose duloxetine over venlafaxine XR when pain was coexistent with depression.

Both duloxetine and venlafaxine XR are to be used with caution in patients with a history of mania, a diagnosis of bipolar disorder and/or seizures. Our results suggested that when depressed patients were on anticonvulsants prior to initiating treatment with venlafaxine XR or duloxetine, their odds of receiving venlafaxine XR were 30% lower than duloxetine. This finding was also consistent with those from Ye et al. [14] study. Anticonvulsants can be used to treat a variety of conditions including seizure, bipolar disorder, and pain conditions such as fibromyalgia and DPN. Although we did not capture the underlying conditions for which anticonvulsants were prescribed, the most frequently used anticonvulsants were gabapentin, pregabalin, lamotrigine, solium valproate, and carbamazepine. Besides treating epilepsy, most of these medications are often used in managing pain such as neuropathic pain and fibromyalgia [19–21]. This may explain the higher odds of initiating duloxetine among patients with pre-period use of anticonvulsants.

Venlafaxine XR has additional approved indications in the UK including generalized anxiety disorder, social anxiety disorder, and panic disorder. Duloxetine is now indicated for generalized anxiety disorder, but the indication was not approved until August 2008, which was at the close of our study period. This may explain, in part, our findings that patients with anxiety disorders had a higher likelihood of initiating treatment with venlafaxine XR than duloxetine. Given that clinical trials show noninferiority in terms of clinical efficacy and tolerability between duloxetine and venlafaxine XR in the treatment of patients with generalized anxiety disorders [22], we may see an increasing use of duloxetine to treat patients with MDD and generalized anxiety disorders in the future. Finally, our study found that patients who were on antihyperlipidemics were more likely to initiate duloxetine than venlafaxine XR. Similarly, the Shi et al. [13] study reported pre-index diagnosis of dyslipidemia as a significant predictor of initiating duloxetine comparing to nonduloxetine antidepressants.

Interpreting the results from this study is subject to several challenges inherent to conducting outcome research studies with the GPRD. As in any administrative data source, we assumed the accuracy of codes entered into the database by providers. Despite a rigorous review process by researchers and coding specialists to identify Read/OXMIS codes for the clinical conditions and healthcare utilization events of interest, it is possible that some relevant codes were missed while others may have been included erroneously. Any such omissions or errors could impact the accuracy of the results presented, but the impact across cohorts should be consistent. As mentioned in Section 2, chronic conditions may not be coded as frequently as they are seen. As a remedy, we extended the pre-period for the evaluation of chronic conditions, thereby increasing the likelihood of their capture. We also identified relevant medication use, which is sometimes a better indicator of comorbidity than diagnoses alone. For example, the rate of diabetes in the duloxetine cohort was 6.6% during the 36-month pre-period using Read/OXMIS codes, but 7.9% of patients had a prescription for an antidiabetic medication in the 12 months before the index date. While these limitations may have hindered us from accurately reporting the rates of comorbid conditions, the impact on both treatment cohorts is expected to be equal.

Pharmaceutical treatment in this study was evaluated solely on prescription orders recorded by general practitioners. Patients may not fill the prescriptions or take the medications as prescribed, a factor that cannot be assessed in the GPRD. Thus, evaluations of prescription medication use reflect how the medications were prescribed, but not necessarily how patients actually took the medications. Prescriptions provided in a hospital or secondary care setting including non-GP practitioners, as well as over-the-counter medications were not captured. The GPRD is generalizable to the UK population receiving primary care. But it does not include the homeless, the incarcerated, members of the armed forces, or individuals who obtain care in private practices. Thus, our study results are not generalizable to the entire UK population.

## 4. Conclusions

Based on a total of 2,195 patients who newly initiated duloxetine or venlafaxine XR, our study suggested that older age, preexisting unexplained pain, anxiety disorders, and respiratory disease, as well as pre-period use of opioid analgesics, antihyperlipidemics, and anticonvulsants were significant predictors of the initiation of duloxetine versus venlafaxine XR. In the UK primary care system, treatment choice of duloxetine or venlafaxine XR appears to be driven by patient-specific mental and medical health characteristics with general practitioners favoring duloxetine over venlafaxine XR when pain conditions coexist with depression.

## Figures and Tables

**Table 1:** Demographic characteristics of patients Initiating duloxetine or venlafaxine XR.

	Duloxetine		Venlafaxine XR		*P* value
	*n*/mean	%/SD	*n*/mean	%/SD	
Number of patients (*n*)	909		1,286		
Female (*n*, %)	615	67.7%	829	64.5%	0.120
Age (*n*, %)					<0.001
18–34	177	19.5%	345	26.8%	<0.001
35–44	206	22.7%	333	25.9%	0.083
45–54	194	21.3%	254	19.8%	0.362
55–64	156	17.2%	193	15.0%	0.174
65+	176	19.4%	161	12.5%	<0.001
Age (mean, SD)	49.6	16.5	45.5	16.1	<0.001

**Table 2:** Pre-period clinical characteristics of patients initiating duloxetine or venlafaxine XR.

	Duloxetine		Venlafaxine XR		*P* value
	*n*	%	*n*	%	
Diagnosed pain conditions^1^					
Diabetic peripheral neuropathy	3	0.3%	2	0.2%	0.398
Fibromyalgia	48	5.3%	46	3.6%	0.052
Osteoarthritis	62	6.8%	74	5.8%	0.307
Chronic low-back pain	14	1.5%	4	0.3%	0.002
Unexplained pain	413	45.4%	431	33.5%	<0.001
Diagnosed psychiatric conditions^1^					
Anxiety disorders	190	20.9%	315	24.5%	0.049
Attention deficit hyperactivity disorder	0	0.0%	2	0.2%	0.234
Schizophrenia	2	0.2%	6	0.5%	0.345
Bipolar disorder	8	0.9%	10	0.8%	0.793
Alcohol/drug dependence	43	4.7%	69	5.4%	0.505
Other diagnosed conditions^1^					
Diseases of the circulatory system	207	22.8%	204	15.9%	<0.001
Diseases of the respiratory system	470	51.7%	571	44.4%	<0.001
Diseases of the digestive system	317	34.9%	378	29.4%	0.007
Sleep disorders	95	10.5%	133	10.3%	0.934
Diabetes	60	6.6%	36	2.8%	<0.001
Healthcare utilization^2^					
Inpatient admission	188	20.7%	224	17.4%	0.054
Accident and emergency Visits	207	22.8%	245	19.1%	0.034

^1^Comorbid conditions were evaluated during 36-month pre-index period.

^2^Healthcare utilization was evaluated during 12-month pre-index period.

**Table 3:** Pre-period medication use^1^among patients initiating duloxetine or venlafaxine XR.

	Duloxetine		Venlafaxine XR		*P* value
	*n*	%	*n*	%	
Antidepressant—SNRI	16	1.8%	10	0.8%	0.036
Antidepressant—TCAs	228	25.1%	273	21.2%	0.034
Antidepressant—MAOIs	8	0.9%	4	0.3%	0.075
Antidepressant—SSRI	679	74.7%	985	76.6%	0.307
Antidepressant—Other	170	18.7%	236	18.4%	0.835
Anxiolytics/hypnotics/muscle relaxants^2^	406	44.7%	530	41.2%	0.107
Antipsychotics	135	14.9%	201	15.6%	0.618
Stimulants	12	1.3%	11	0.9%	0.292
Antimanics	19	2.1%	21	1.6%	0.43
Anticonvulsants	86	9.5%	66	5.1%	<0.001
Analgesics—opioids^3^	329	36.2%	305	23.7%	<0.001
Analgesics—non-opioids	427	47.0%	436	33.9%	<0.001
Analgesics—migraine medications	57	6.3%	86	6.7%	0.697
Cardiovascular Disease Medications	340	37.4%	324	25.2%	<0.001
Antihyperlipidemics	189	20.8%	124	9.6%	<0.001
Antihypertensives	230	25.3%	186	14.5%	<0.001
Other medications	108	11.9%	120	9.3%	0.054
Thyroid hormones	68	7.5%	67	5.2%	0.029
Antidiabetics	72	7.9%	48	3.7%	<0.001
GI medications	381	41.9%	429	33.4%	<0.001
Respiratory medications	157	17.3%	195	15.2%	0.185

^1^Medications were evaluated during 12-month pre-index period.

^2^Included sedatives, hypnotics, muscle relaxants, and all benzodiazepines, some of which are used to treat conditions other than anxiety or insomnia.

^3^Included all medications under BNF “Opioid analgesics” except codeine formulations used to treat diarrhea and migraine medications that contain codeine (e.g., Migraleve).
